# Mortality Benefit of Convalescent Plasma in COVID-19: A Systematic Review and Meta-Analysis

**DOI:** 10.3389/fmed.2021.624924

**Published:** 2021-04-09

**Authors:** Vikas Bansal, Kiran S. Mahapure, Ishita Mehra, Abhishek Bhurwal, Aysun Tekin, Romil Singh, Ishita Gupta, Sawai Singh Rathore, Hira Khan, Sohiel Deshpande, Shivam Gulati, Paige Armaly, Mack Sheraton, Rahul Kashyap

**Affiliations:** ^1^Department of Anaesthesiology and Critical Care Medicine, Mayo Clinic, Rochester, MN, United States; ^2^Senior Resident, Department of Plastic Surgery, KAHER J. N. Medical College, Belgaum, India; ^3^Department of Internal Medicine, North Alabama Medical Center, Florence, AL, United States; ^4^Department of Gastroenterology and Hepatology, Rutgers Robert Wood Johnson School of Medicine, New Brunswick, NJ, United States; ^5^Department of Internal Medicine, Metropolitan Hospital, Jaipur, India; ^6^Department of Internal Medicine, Dr. Rajendra Prasad Government Medical College, Tanda, India; ^7^Department of Internal Medicine, Dr. Sampurnanand Medical College, Jodhpur, India; ^8^Department of Internal Medicine, Riphah International University Islamic International Medical College, Rawalpindi, Pakistan; ^9^Department of Internal Medicine, Maharashtra Institute of Medical Education and Research, Pune, India; ^10^Department of Internal Medicine, Adesh Institute of Medical Sciences and Research, Bathinda, India; ^11^Department of Internal Medicine, University of the West Indies, Nassau, Bahamas; ^12^Department of Emergency Medicine, Trinity West Medical Center MSOPTI EM Program, Steubenville, OH, United States

**Keywords:** COVID-19, SARS-CoV 2, mortality, plasma therapy, systemic review and meta-analysis, convalescent plasma

## Abstract

**Importance/Background:** With a scarcity of high-grade evidence for COVID-19 treatment, researchers and health care providers across the world have resorted to classical and historical interventions. Immunotherapy with convalescent plasma (CPT) is one such therapeutic option.

**Methods:** A systematized search was conducted for articles published between December 2019 and 18th January 2021 focusing on convalescent plasma efficacy and safety in COVID-19. The primary outcomes were defined as mortality benefit in patients treated with convalescent plasma compared to standard therapy/placebo. The secondary outcome was pooled mortality rate and the adverse event rate in convalescent plasma-treated patients.

**Results:** A total of 27,706 patients were included in the qualitative analysis, and a total of 3,262 (2,127 in convalescent plasma-treated patients and 1,135 in the non-convalescent plasma/control group) patients died. The quantitative synthesis in 23 studies showed that the odds of mortality in patients who received plasma therapy were significantly lower than those in patients who did not receive plasma therapy [odds ratio (OR) 0.65, 95% confidence interval (CI) 0.53–0.80, *p* < 0.0001, *I*^2^ = 15%). The mortality benefit remains the same even for 14 trials/prospective studies (OR 0.59, 95% CI 0.43–0.81, *p* = 0.001, *I*^2^ = 22%) as well as for nine case series/retrospective observational studies (OR 0.78, 95% CI 0.65–0.94, *p* = 0.01, *I*^2^ = 0%). However, in a subgroup analysis for 10 randomized controlled trials (RCTs), there was no statistically significant reduction in mortality between the CPT group compared to the non-CPT group (OR 0.76, 95% CI 0.53–1.08, *p* = 0.13, *I*^2^ = 7%). Furthermore, the sensitivity analysis of 10 RCTs, excluding the study with the highest statistical weight, displayed a lower mortality rate compared to that of non-CPT COVID-19 patients (OR 0.64, 95% CI 0.42–0.97, *p* = 0.04, *I*^2^ = 0%). The observed pooled mortality rate was 12.9% (95% CI 9.7–16.9%), and the pooled adverse event rate was 6.1% (95% CI 3.2–11.6), with significant heterogeneity.

**Conclusions and Relevance:** Our systemic review and meta-analysis suggests that CPT could be an effective therapeutic option with promising evidence on the safety and reduced mortality in concomitant treatment for COVID-19 along with antiviral/antimicrobial drugs, steroids, and other supportive care. Future exploratory studies could benefit from more standardized reporting, especially in terms of the timing of interventions and clinically relevant outcomes, like days until discharge from the hospital and improvement of clinical symptoms.

## Highlights

### What we Already Know About This Topic

- COVID-19 is an ongoing global pandemic, for which convalescent plasma has been recommended as a possible therapeutic drug.
- Preliminary clinical trial results propose that there may be a satisfactory safety profile and better clinical outcome for patients treated with convalescent plasma compared with those treated with placebo or were under standard of care; however, data are limited at the current time.

### What This Article Tells Us That Is New

- This systematic review and meta-analysis provides an exhaustive summary of current literature on the efficacy and safety of convalescent plasma use in COVID-19 patients.

## Introduction

The first case of coronavirus was identified in Wuhan, China, at the end of 2019 (1). The World Health Organization (WHO) declared a public health emergency of international concern on 30th January 2020 and a global pandemic on 11th March 2020 (2). The WHO estimates that serious illness occurs in 13.8% of cases and that 6.1% cases are critical (3). As of 3rd February 2021, there have been 104,077,986 confirmed cases of COVID-19, including 2,259,391 deaths, reported worldwide (4).

Severe Acute Respiratory Syndrome Coronavirus 2 (SARS-CoV-2) is an RNA virus that is believed to primarily affect the respiratory tract; however, numerous complications related to systems other than the respiratory system have also been noted (5). Even though certain drugs, such as remdesivir, have been repositioned for emergency use in COVID-19, no particular drugs have yet been identified as an effective treatment of COVID-19. Therefore, various clinical trials are ongoing in search for the best therapy. With a scarcity of high-grade evidence for COVID-19 treatment, researchers and health care providers across the world have resorted to classical and historical interventions. Immunotherapy with convalescent plasma (CPT) is one such therapeutic option.

Convalescent plasma uses have been well-described in various diseases such as severe acute respiratory syndrome (6), Middle East respiratory syndrome coronavirus (6), Ebola virus disease (7), pandemic influenza A (6), and avian-origin influenza A (6), and a neutralizing antibody response directed against the viral S protein of the SARS virus has been reported (8). The antibodies primarily target the trimeric spike (S) surface glycoproteins, which are used by the virus to enter the host cells (9). The antibody thus hinders the ability of the SARS-CoV-ACE2 to enter the host cells and can be detected even 24 months after the onset of infection (9). Subsequently, the Food and Drug Administration (FDA) approved the use of convalescent plasmas for patients with serious or immediately life-threatening COVID-19 infections on 24th March 2020 (10).

One of the first studies demonstrating the benefit of CPT was reported in April 2020 (11). Since then, there has been increasing interest (12, 13), and three inconclusive Cochrane reviews (14–16) revealed that unmatched cohort studies are still the most frequent reports. As the literature around CPT is evolving and newer studies are being reported across the world, we conducted a systematic review and meta-analysis to appraise the currently available data for the clinical usefulness of convalescent plasma for the treatment of COVID-19. Organizing summaries of the available clinical evidence regarding safety and effectiveness from published literature through a systematic review can provide a synopsis of clinical evidence on the potential benefits and adverse events of CPT therapy in critically ill COVID-19 patients.

## Methods

Our study has been performed in accordance with the Preferred Reporting Items for Systematic Reviews and Meta-analyses (PRISMA) statement (17, 18).

### Search Strategy

The search strategy was designed and conducted by the authors (IM, KM, and VB). A systematic search was conducted from COVID-19 inception through 7th August 2020 for full-length articles focusing on the efficacy and safety of convalescent plasma in COVID-19 in three major COVID-19 research article databases, namely, WHO Global Research Database, CDC COVID-19 Research Articles Downloadable Database, and LitCovid database. These databases automatically gathered for articles related to COVID-19. Other literature sources such as the Eurosurveillance, China CDC Weekly, Homeland Security Digital Library, ClinicalTrials.gov, bioRxiv (preprints), medRxiv (preprints), chemRxiv (preprints), and SSRN (preprints) were searched as well. The search strategy consisted of a combination of keywords such as “Convalescent Plasma, Plasma therapy, COVID-19, SARS-CoV 2, Mortality, Systemic, Review, Meta-analysis” across the combined COVID-19 databases. After a thorough search was performed, full-length articles meeting the inclusion criteria were evaluated. All titles and abstracts were identified by the authors and screened to accrue potentially eligible studies. A manual search of the references of the included studies was also performed to supplement the electronic search. Then, the same reviewers (AT, IG, KM, PA, RS, and SG) independently assessed all selected full-text manuscripts for eligibility.

### Eligibility Criteria

The specific inclusion criteria for the systematic review and meta-analysis were as follows: (1) all RCTs or prospective studies or retrospective studies in hospitalized patients with COVID-19, (2) the use of plasma as therapy for COVID-19, (3) all studies with information available to evaluate the incidence of mortality in COVID-19 patients with plasma use [number of events, sample size, odds ratio (OR), and confidence interval (CI)], and (4) full-text articles. Thus, reviewed studies included in our analysis were RCTs and prospective and retrospective studies evaluating the outcomes of plasma therapy in COVID-19 patients. Studies focusing on patients <18 years of age, focusing on pregnant females, and limited to particular comorbidities and organ dysfunctions were excluded to avoid selection bias. We also excluded case reports from our systematic review.

### Data Extraction

**Figure 1:**
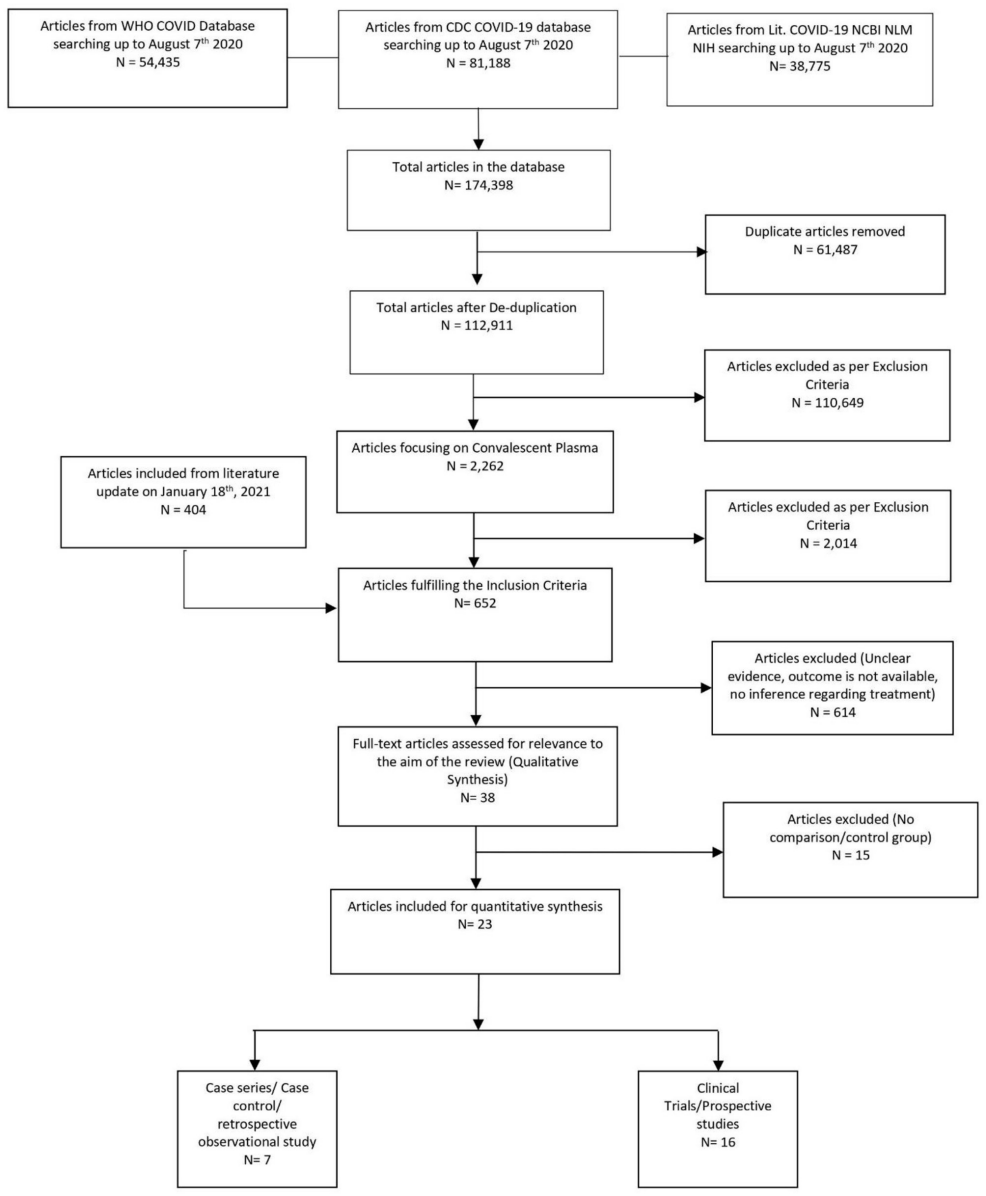
PRISMA study flow diagram.

Once the studies met the inclusion criteria, four reviewers (AT, HK, IG, IM, KM, RS, or SD) independently reviewed and abstracted data for mortality rate and adverse event rate for each eligible study (Figure 1). If there were multiple reports stemming from a specific study database, data from the most robust study were extracted, with other studies contributing toward the bibliography. Subsequently, the data were collected and tabulated using Microsoft Excel. The included data were checked for accuracy by AB, KM, IG, and VB. The reviewers sorted the data separately in all stages of study collection, data extraction, and quality assessment. All discrepancies found between two reviewers were resolved with consensus and inputs from other authors.

### Study Characteristics and Quality Assessment

Randomized trial and prospective studies were evaluated using the Cochrane risk-of-bias tool (19), and the correlation of quality measures with estimates of treatment effects in the meta-analyses of RCTs (20) was used for quality assessment of the same. We used the NIH Quality Assessment Tool for Case Series Studies (21) and the Newcastle–Ottawa Scale (NOS) (22) for case–control or non-randomized retrospective cohort studies. For each non-randomized study, we assessed the study design and content. The studies were then graded using a “star system” on the basis of (1) the selection of the study groups, (2) the comparability of the groups, and (3) the ascertainment of the outcome of interest. Quality assessments were also conducted independently, and discrepancies were resolved by consensus.

### Outcome Measures

All the studies describing the outcomes of plasma therapy in patients with COVID-19 were analyzed in detail. Primary outcomes were mortality benefits for patients on CPT in COVID-19. The mortality rate was evaluated in comparison to that of the control group (placebo or non-CPT). The defined secondary outcome was the pooled mortality rate and pooled adverse event rate.

### Quantitative Data Synthesis

Primary outcomes were analyzed by the Review Manager (RevMan) computer program, version 5.4 for Windows (23), and the Comprehensive Meta-Analysis software package (BioStat, Englewood, NJ, USA) (24) was used for calculating the mortality and adverse event rates. The final pooled risk estimates were obtained using random effects models (25). Raw data for outcomes and non-events from each study were used to calculate crude OR with respective 95% CI for each study. The Cochrane *Q* and the *I*^2^ statistics were calculated to assess heterogeneity between studies (25, 26). *p* < 0.10 for chi-square tests and *I*^2^ <20% were interpreted as low-level heterogeneity. We planned to perform a subgroup analysis by study design (trial/prospective studies and observational) to decrease burden of selection bias of the observational studies. It is expected that the estimates from observational studies will be more overestimated than those from RCTs (26). Furthermore, we planned to conduct a sensitivity analysis for randomized trials in trial/prospective studies to check for robustness of the results. The probability of publication bias was assessed using funnel plots and Egger's tests.

## Results

The initial library search identified potentially relevant citations from the WHO Global Research Database, CDC COVID-19 Research Articles Downloadable Database, and LitCovid PubMed database comprising 174,398 articles. Subsequently, 61,487 duplicates were removed. Out of the remaining 112,911 articles, a total of 2,262 focused on convalescent plasma. A total of 2,014 articles were excluded after title and abstract reviews due to not having patient data. We added 404 articles during literature update on 18th January 2021 in the initial literature search. The remaining 652 manuscripts were scrutinized further, and 615 were further excluded because of unclear evidence and non-relevance to the objective of the manuscript. Thus, 38 studies (11, 12, 27–62) were included in their entirety, as shown in Table 1. The PRISMA flowchart is shown in Figure 1.

**Table 1A:** Study characteristics.

**References**	**Type of study**	**Dose of convalescent plasma**	**Hospital length of stay**	**Antibodies titer**	**Viral shedding**	**Concomitant treatment with CPT**	**Adverse events**
Abolgashemi et al. (27)	Trial	500 ml	9.54 days	N/D	N/D	Lopinavir/ritonavir, hydroxychloroquine and an anti-inflammatory agent	Transient mild fever and chill in one patient
Agarwal et al. (28)	Trial	200 ml	14 days	N/D	N/D	Methylprednisolone, prednisone, azithromycin, hydroxychloroquine, lopinavir, and ritonavir	Pain in the local infusion site, chills, nausea, bradycardia, and dizziness reported in one patient each. Fever and tachycardia reported in three patients each. Dyspnea and intravenous catheter blockage noted in two patients each. Mortality assessed as possibly related to convalescent plasma (CP) transfusion in three patients
Ahn et al. (29)	Case series	500 ml, into two doses	28 days	N/D	N/D	Lopinavir/ritonavir, hydroxychloroquine, and methylprednisolone	No adverse reactions were observed
Altuntas et al. (54)	Case–control	200–600 ml	17–18 days	N/D	N/D	Antiviral azithromycin	N/A
Avenado-Sola et al. (62)	Multicenter randomized clinical trial	250–300 ml	N/A	N/A	N/A	Yes	Six in CP, seven in standard of care (SOC)
Bajpai et al. (59)	Open-labeled randomized controlled trial (RCT)	500 ml	14 days	>80	N/A	Hydroxychloroquine, azithromycin, and oseltamivir	Mild urticaria in one patient each of CP and fresh frozen plasma (FFP) arms
Donato et al. (30)	Trial with matched cohort study	200–500 ml	N/D	1:1,000–10,000 to >1:10,000 in some patients	N/D	Hydroxychloroquine, steroids, remdesivir, azithromycin, and tocilizumab	Mild rash in one patient
Duan et al. (11)	Case control	200 ml	N/D	1:640	N/D	Arbidol, remdesivir, and interferon-alpha	Facial red spot in one patient
Gharbaran et al. (31)	RCT	300 ml	N/D	>1:20	N/D	Chloroquine, azithromycin, lopinavir/ritonavir, tocilizumab, and anakinra	No adverse reactions were observed
Liu et al. (35)	Case–control	250 ml	N/D	≥1:320	N/D	Azithromycin, broad-spectrum antibiotics, hydroxychloroquine, antivirals, corticosteroids, interleukin-6 inhibitors, and therapeutic anticoagulation	No adverse reactions were observed
Hartman et al. (32)	Single-arm trial	N/D	12 days	N/D	N/D	Data unavailable	N/D
Hegerova et al. (33)	Case–control	N/D	15 days	N/D	N/D	Azithromycin and hydroxychloroquine	No adverse reactions were observed
Joyner et al. (12)	Clinical trial	200–500 ml	N/D	N/D	N/D	N/D	Transfusion reactions (*n* = 78; <1%), thromboembolic or thrombotic events (*n* = 113; <1%), and cardiac events (*n* = 677)
Karekadavath et al. (51)	Case series	200 ml	22–43 days	N/D	20–42 days	Remdesivir and ribavirin	N/D
Li et al. (34)	Trial	4–13 ml/kg of recipient body weight	7–28 days	N/D	N/D	Antiviral, interferon, Chinese herbal medicine, antibacterial, antifungal, steroids, and human immunoglobulin	Seen in two patients
Libster et al. (58)	Double-blind placebo RCT	250 ml	N/A	>1:1,000	N/A	N/A	N/A
Maor et al. (36)	Prospective cohort	200 ml	N/D	≥1:80	N/D	Tocilizumab	Rash in one patient
Martinez-Resendez et al. (37)	Case series	250 ml	22.5 days	> 1:100	N/D	Chloroquine/hydroxychloroquine, lopinavir/ritonavir, azithromycin, and ceftaroline	No adverse reactions were observed
Erkurt et al. (52)	Trial	200 cm^3^	7 days	>1:640	N/D	N/D	No adverse reactions were observed
Olivares-Gazca et al. (38)	Prospective non-randomized pilot trial	200 ml	N/D	N/D	N/D	Steroids, hydroxychloroquine, azithromycin, tocilizumab, and lopinavir/ritonavir	No adverse reactions were observed
Omrani et al. (53)	Retrospective cohort	400 ml	N/D	N/D	N/A	Hydroxychloroquine, azithromycin, lopinavir, ritonavir, and tocilizumab	77
Pappa et al. (39)	Phase II trial	200–233 ml	21 days	N/D	N/D	Hydroxychloroquine, remdesivir, lopinavir/ritonavir, methylprednisolone, dexamethasone, hydrocortisone, tocilizumab, heparin (UFH/LMWH), azithromycin, and intravenous immunoglobulin	No adverse reactions were observed
Pei et al. (40)	Case series	Data unavailable	26–36 days	N/D	12–29 days	Data unavailable	Severe anaphylactic shock
Perotti et al. (41)	Trial	250–300 ml	N/D	>1:160	N/D	Lopinavir/ritonavir, darunavir/ritonavir, darunavir/cobicistat, antibiotics, hydroxychloroquine, and anticoagulant	Chills and fever during transfusion, anaphylaxis/hypersensitivity, transfusion acute lung injury, urticaria
AlQahtani et al. (60)	Open-labeled RCT	400 ml	NA	N/A	N/A	Hydroxychloroquine, ribavirin, lopinavir/ritonavir, and tocilizumab	One transient desaturation, one diarrhea, vomiting
Rasheed et al. (42)	Randomized trial	N/D	21 days	N/D	N/D	N/D	Mild skin redness and itching in one patient
Ray et al. (61)	Open-labeled phase II RCT	200 ml	23 for SOC vs. 17 for CPT	N/A	N/A	Hydroxychloroquine, azithromycin, ivermectin, doxycycline, and corticosteroids	N/A
Rogers et al. (55)	Matched cohort study	One unit	N/D	N/D	N/D	Corticosteroid and remdesivir	N/D
Salazar et al. (43)	Trial with matched cohort study	One or two units of COVID-19 convalescent plasma		≥1:1,350	N/D	Dexamethasone and hydrocortisone	N/D
Shen et al. (44)	Case series	400 ml	Average 46 days	1:1,000	N/D	Lopinavir/ritonavir, methylprednisolone, arbidol, favipiravir, and interferon-alpha	No adverse event mentioned
Simonovich et al. (56)	Trial	500 ml	30 days	1:3,200	N/D	Antiviral agents and glucocorticoids	No adverse reactions were observed
Tan et al. (45)	Case series	400 ml	17 days for one patient	N/D	16 and 49 days	Antiviral medicines and Chinese traditional medicines	N/D
Wang et al. (46)	Case series	200 ml	51 days	N/D	N/D	Hydroxychloroquine, methylprednisolone, lopinavir, ritonavir, tocilizumab, low-molecular-weight heparin, azithromycin, and oseltamivir	No adverse reactions were observed
Xia et al. (47)	Case–control	200–1,200 ml	22 days	N/D	N/D	N/D	Minor allergic reactions (pruritus or erythema) in three patients
Ye et al. (48)	Case series	200 ml	15–24 days	N/D	N/D	Arbidol and levofloxacin	No adverse reactions were observed
Yoon et al. (57)	Retrospective cohort	200 ml	N/A	>1:1,000	N/A	Corticosteroids	N/A
Zeng et al. (49)	Case–control	300 (200–600) ml	N/D	N/D	23.5 days	Glucocorticoid and traditional Chinese medicine	No adverse reactions were observed
Zhang et al. (50)	Case series	200–2,400 ml	21–41 days	N/D	N/D	Lopinavir/ritonavir, methylprednisolone, arbidol, favipiravir, interferon-alpha, and oseltamivir	No adverse reactions were observed

*N/A, not available; N/D, not defined/not mentioned*.

### Study Characteristics

A total of 38 studies (11, 12, 27–62) were included in the qualitative analysis (Tables 1A,B). Out of which, 23 studies (11, 27, 28, 30, 31, 33–35, 41–43, 47, 49, 53–62) compared the mortality in convalescent plasma-treated patients vs. that in patients treated by standard therapy/placebo. Out of 14 trials/prospective studies, 10 trials (28, 31, 34, 42, 56, 58–62) conducted proper randomization, and six trials/prospective studies matched with the cohort retrospectively (27, 30, 41, 43). Zhang et al. (50) concluded that seroconversion occurred in 5–24 days, while Zeng et al. (49) mentioned that all six patients tested negative within 3 days of starting convalescent plasma. Tan et al. (45) evaluated the viral shedding period in convalescent plasma-treated patients, which was 16–46 days. Joyner et al. conducted the largest interventional case study with 20,000 convalescent plasma-treated patients and evaluated the safety profile (12).

**Table 1B:** Study characteristic outcomes.

**References**	**Mortality CPT arm**	**Total CPT patient**	**Mortality Non-CPT arm**	**Total Non-CPT patient**	**Adverse event CPT**	**ICU admission**	**ARDS**	**Mechanical ventilation**	**ECMO**
Abolgashemi et al. (27)	17	115	18	74	1	N/A	N/A	8	0
Agarwal et al. (28)	34	235	31	229	9	N/A	N/A	18	0
Ahn et al. (29)	0	2			0	2	2	2	0
Altuntas et al. (54)	219	888	245	888	0	21	N/A	926	N/A
Avenado-Sola et al. (62)	0	38	4	43	6	N/A	N/A	N/A	N/A
Bajpai et al. (59)	3	14	1	15	1	N/A	N/A	4	0
Donato et al. (30)	11	47	565	1,340	1	N/A	N/A	15	0
Duan et al. (11)	0	10	3	10	1	2	2	3	0
Gharbaran et al. (31)	6	43	11	43	0	31	31	31	5
Liu et al. (35)	5	39	38	156	0	4	4	4	0
Hartman et al. (32)	4	31			0	6	15	10	0
Hegerova et al. (33)	2	20	6	20	4	6	6	6	0
Joyner et al. (12)	1,711	20,000			1,282	11,560	9,729	6,864	0
Karekadavath et al. (51)	0	4			0	4	4	1	0
Li et al. (34)	8	51	12	50	2	29	N/A	14	14
Libster et al. (58)	2	80	4	80	0	8	7	6	0
Maor et al. (36)	9	49			1		28	28	0
Martinez-Resendez et al. (37)	0	8			0	8	8	5	0
Erkurt et al. (52)	6	26	0	0	0	0	0	6	0
Olivares-Gazca et al. (38)	2	10			0	10	5	5	0
Omrani et al. (53)	1	40	5	40	N/A	80	N/A	69	0
Pappa et al. (39)	0	9			1	9	9	2	0
Pei et al. (40)	0	3			1	0	0	0	0
Perotti et al. (41)	3	46	7	23	4	16	43	7	2
AlQahtani et al. (60)	1	20	2	20	3	N/A	N/A	10	0
Rasheed et al. (42)	1	21	8	28	2	21	21	21	0
Ray et al. (61)	10	40	14	40	0	N/A	N/A	N/A	N/A
Rogers et al. (55)	8	64	28		177	85	0	28	0
Salazar et al. (43)	5	136	19	251	0	161	21	21	1
Shen et al. (44)	0	5			0	5	5	5	1
Simonovich et al. (56)	25	228	12	105	220	8	0	19	0
Tan et al. (45)	0	2			0	0	0	0	0
Wang et al. (46)	3	5			0	5	5	5	0
Xia et al. (47)	3	138	59	1,430	0	3	22	28	2
Ye et al. (48)	0	6			0	1	0	4	0
Yoon et al. (57)	23	73	28	73	0	N/A	N/A	18	0
Zeng et al. (49)	5	6	14	15	0	6	6	5	1
Zhang et al. (50)	0	4			0	4	4	2	2

*N/A, not available/not mentioned*.

### Primary Outcome

#### Mortality Comparison Between Plasma Therapy and Placebo

**Figure 2:**
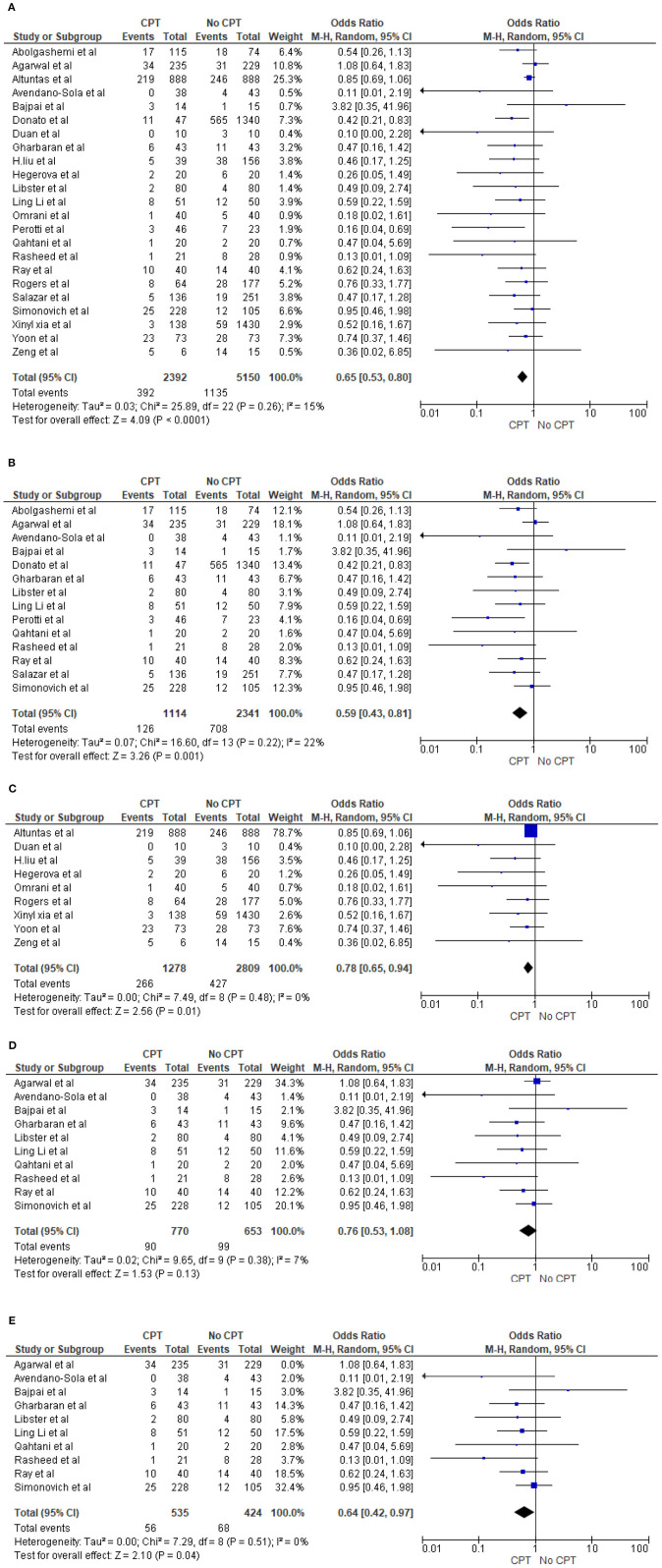
**(A)** Overall comparison of mortality rate in patients on CPT vs. non-CPT treatment. **(B)** Subgroup analysis of mortality rate in patients on CPT vs. non-CPT treatment (trial/prospective studies). **(C)** Subgroup analysis of mortality rate in patients on CPT vs. non-CPT treatment (observational). **(D)** Sensitivity analysis for mortality rate in patients on CPT vs. non-CPT treatment (trial/prospective studies) true randomized controlled trial (removed pseudorandomized or trial with matched cohort). **(E)** Sensitivity analysis for mortality rate in patients on CPT vs. non-CPT treatment (trial/prospective studies) true randomized controlled trial [removed Agarwal et al. (28)].

Twenty-three studies reported the mortality rate in COVID-19 patients on plasma and non-CPT therapy (11, 27, 28, 30, 31, 33–35, 41–43, 47, 49, 53–62). This yielded a sample size of 7,542 patients, with 2,392 patients on plasma therapy and 5,150 patients in the control group. In the CPT therapy cohort, 392 patients died, while 1,135 patients died in the placebo/non-CPT cohort. The meta-analysis of these mortality rates showed that the odds of mortality on plasma therapy were significantly lower than those in patients who did not receive plasma therapy (OR 0.65, 95% CI 0.53–0.80, *p* < 0.0001, *I*^2^ = 15%). This is shown in a Forrest plot (Figure 2A). We performed a subgroup analysis by study designs and observed similar mortality benefits in 14 trial/prospective studies (27, 28, 30, 31, 34, 41–43, 56, 58–62) (OR 0.59, 95% CI 0.43–0.81, *p* = 0.001, *I*^2^ = 22%) (Figure 2B) as well as for nine case series/retrospective observational studies (11, 33, 35, 47, 49, 53–55, 57) (OR 0.78, 95% CI 0.65–0.94, *p* = 0.01, *I*^2^ = 0%) (Figure 2C). However, during the sensitivity analysis of 10 randomized trials (28, 31, 34, 42, 56, 58–62), no statistically significant reduction of COVID-19 deaths was shown (OR 0.76, 95% CI 0.53–1.08, *p* = 0.13, *I*^2^ = 7%) (Figure 2D). Agarwal et al. (28) demonstrated a different effect and had a large statistical weight (34.3%). Therefore, a sensitivity analysis was performed by excluding the study by Agarwal et al. (28). This revealed a significant reduction in the odds of mortality with COVID-19 (OR 0.64, 95% CI 0.42–0.97, *p* = 0.04, *I*^2^ = 0%) (Figure 2E).

### Secondary Outcome

#### Pooled Mortality Rate

**Figure 3:**
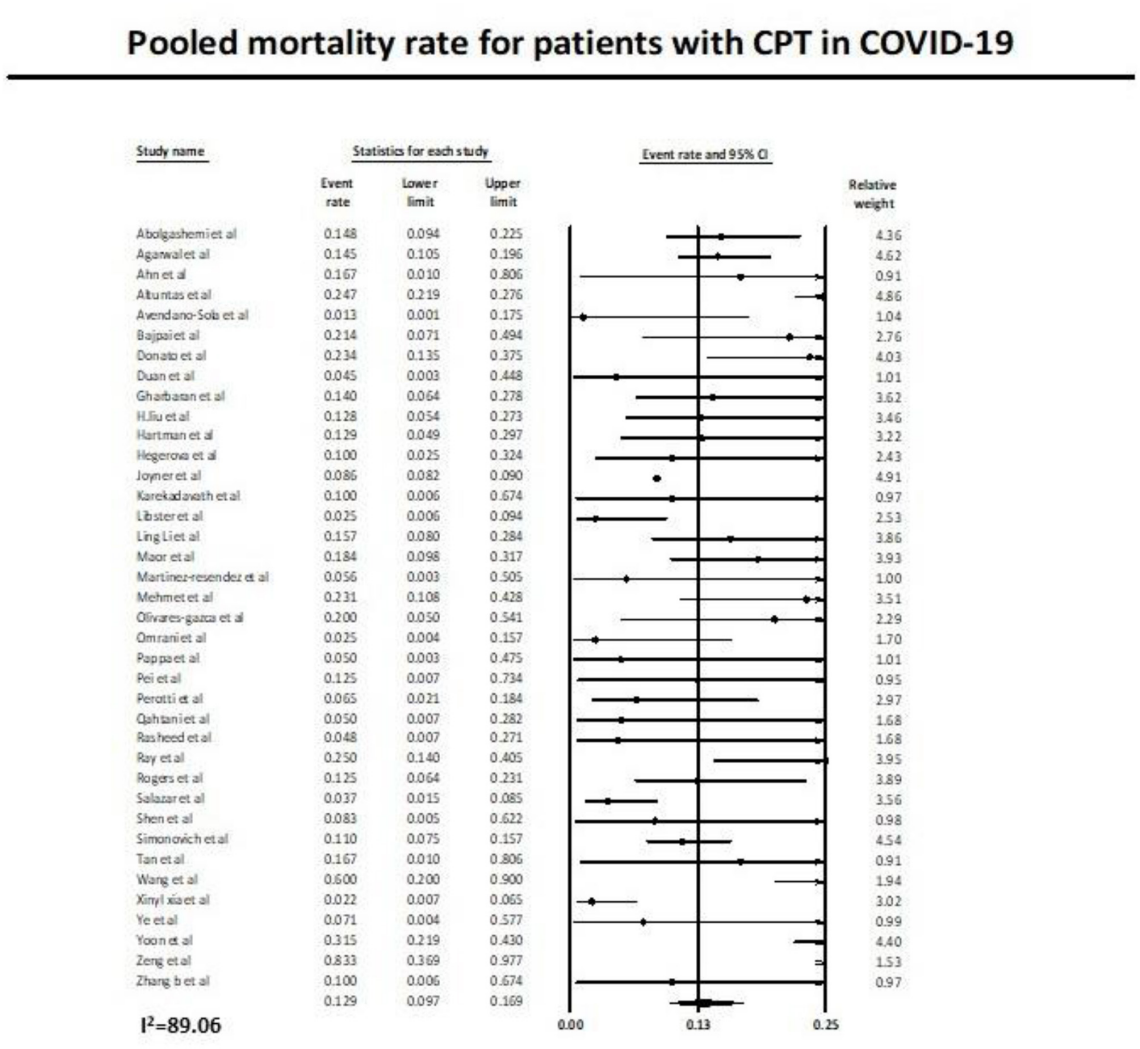
Pooled mortality rate with use of CPT in COVID-19.

Thirty-eight studies (11, 12, 27–62) reported the mortality rate in COVID-19 patients on plasma therapy, as shown in Figure 3. A total of 22,556 patients with CPT were included in the analysis, of which a total of 2,127 patients died. This yielded a pooled post CPT mortality rate of 12.9% (95% CI 9.7–16.9) with a substantial amount of heterogeneity (*I*^2^ = 89.6) in the analysis (Figure 3).

#### Pooled Adverse Event Rate

**Figure 4:**
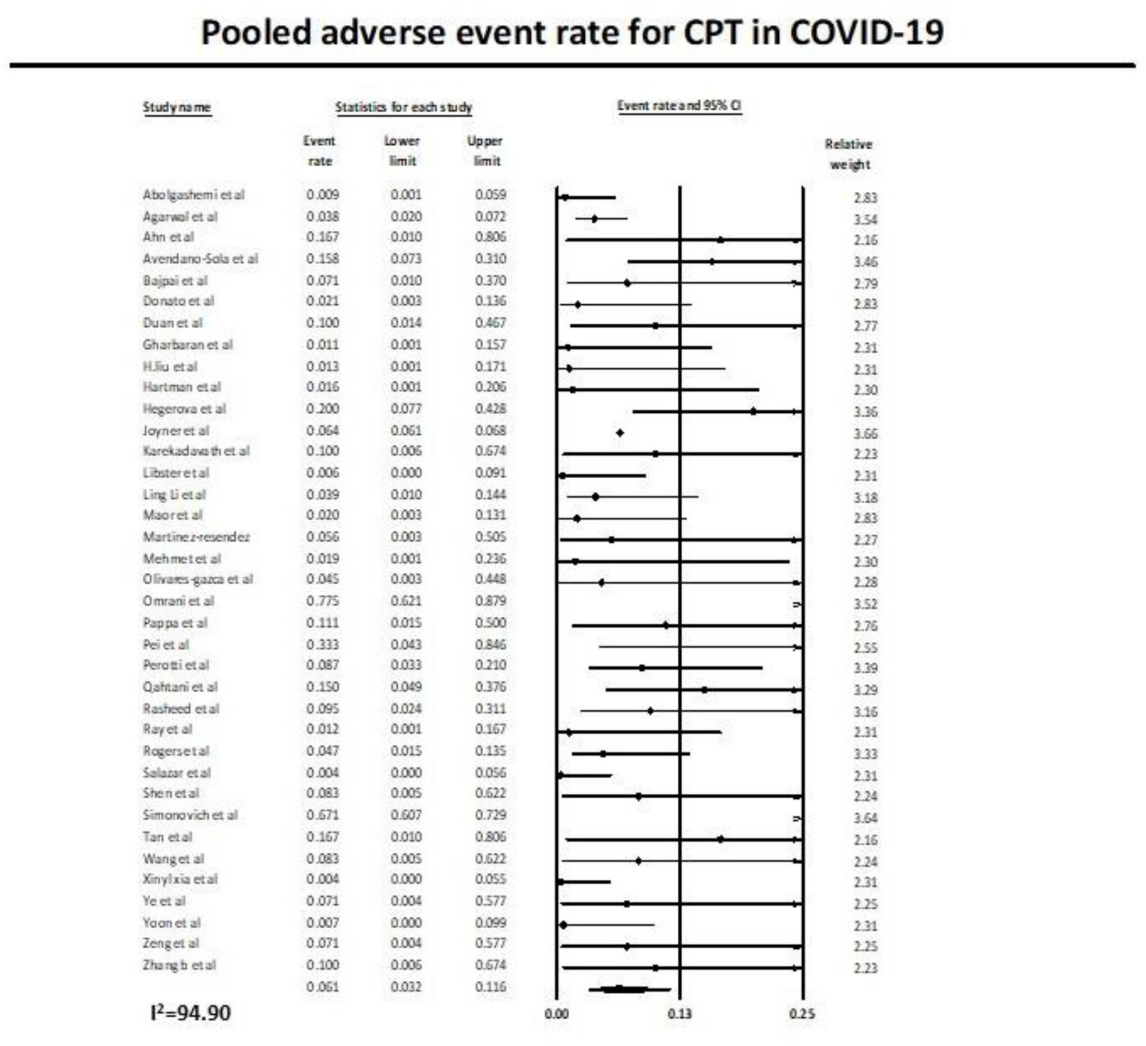
Pooled adverse event rate with use of CPT in COVID-19.

Similarly, 37 studies (11, 12, 27–53, 55–62) reported the adverse event rate in COVID-19 patients on plasma therapy, as shown in Figure 4. A total of 21,668 patients with CPT were included in the analysis, of which a total of 1,506 patients had adverse events. This yielded a pooled adverse event rate of 6.1% (95% CI 3.2–11.6) with significant heterogeneity in the analysis (*I*^2^ = 94.9) (Figure 4).

### Risk-of-Bias Assessment

Two authors (KM and AT) independently assessed the risk of bias of each study included. All disagreements were discussed with all the authors, and decisions were made via a consensus. The Cochrane tool for risk of bias (19) was used for RCTs (Table 2A), and the correlation of quality measures with estimates of treatment effects in meta-analyses of RCTs (20) was used for quality assessment of the same (Table 2B). Non-randomized studies were evaluated using the NOS for the case–control/cohort (22) (Tables 2C,D) and the NIH Quality Assessment Tool for Case Series Studies (21) (Table 2E). Quality assessments were conducted independently, and discrepancies were resolved by consensus. Overall, risk-of-bias assessment showed that the included studies had low to medium risk of bias.

Table 2AAssessment of the trials included in the study.

**Table:** 

**References**	**Abolgashemi et al. (27)**	**Agarwal et al. (28)**	**AlQahtani et al. (60)**	**Avenado-Sola et al. (62)**	**Bajpai et al. (59)**	**Donato et al. (30)**	**Erkurt et al. (52)**	**Hartman et al. (32)**	**Gharbaran et al. (31)**	**Joyner et al. (12)**
Study question well-defined in introduction/methods	Yes	Yes	Yes	Yes	No	Yes	Yes	Yes	Yes	Yes
Study question well-defined anywhere in the article	Yes	Yes	Yes	Yes	No	Yes	Yes	Yes	Yes	Yes
Placebo control	No	No	No	No	No	No	No	No	No	No
Appropriate outcome studied	Yes	Yes	Yes	Yes	Yes	Yes	Yes	Yes	Yes	Yes
Multicenter study	Yes	Yes	Yes	Yes	No	No	No	No	Yes	Yes
Study country	Iran	India	Bahrain	Spain	India	USA	Turkey	USA	Netherlands	USA
Adequate selection criteria	Yes	Yes	Yes	Yes	Yes	Yes	No	Yes	Yes	Yes
Randomization methods described	N/A	Yes	Yes	Yes	Yes	N/A	N/A	N/A	Yes	N/A
Central randomization site	N/A	Yes	N/D	N/D	N/A	N/A	N/A	N/A	N/D	N/A
Allocation concealment	N/D	Yes	No	Yes	Yes	N/D	N/A	N/D	No	N/D
Patients blinded	N/D	No	No	No	No	N/D	N/A	N/D	No	N/D
Caregivers blinded	N/D	No	No	No	No	N/D	N/A	N/D	No	N/D
Outcome assessors blinded	N/D	No	N/D	N/D	N/D	N/D	N/A	N/D	N/D	N/D
Data analysts blinded	N/D	N/D	N/D	N/D	N/D	N/D	N/A	N/D	N/D	N/D
Double blinded	N/D	No	No	No	No	N/D	N/A	N/D	N/D	N/D
Vital statistical measures	Yes	Yes	Yes	Yes	Yes	Yes	Yes	Yes	Yes	Yes
Statistician author or acknowledged	No	Yes	No	Yes	N/D	Yes	No	No	No	Yes
Intention-to-treat analysis	No	Yes	No	No	No	No	No	No	No	No
Power calculation reported	No	Yes	Yes	Yes	N/D	Yes	No	No	Yes	No
Stopping rules described	No	No	No	Yes	N/D	No	No	No	Yes	No
Baseline characteristics reported	Yes	Yes	Yes	Yes	Yes	Yes	Yes	Yes	Yes	Yes
Groups similar at baseline	No	Yes	Yes	N/D	Yes	N/A	N/A	No	No	N/A
Confounders accounted for	Yes	Yes	Yes	No	Yes	Yes	No	Yes	Yes	No
Percentage dropouts	N/A	Yes	N/A	Yes	Yes	N/A	N/A	N/A	N/A	N/A
Reasons for dropout given	N/A	Yes	N/A	Yes	Yes	N/A	N/A	N/A	N/A	N/A
Findings support conclusion	Yes	Yes	Yes	Yes	Yes	Yes	Yes	Yes	Yes	Yes

**Table:** 

**References**	**Li et al**. **(****34****)**	**Libster et al**. **(****58****)**	**Olivares-Gazca et al**. **(****38****)**	**Pappa et al**. **(****39****)**	**Perotti et al**. **(****41****)**	**Rasheed et al**. **(****42****)**	**Ray et al**. **(****61****)**	**Salazar et al**. **(****43****)**	**Simonovich et al**. **(****56****)**
Study question well-defined in introduction/methods	Yes	Yes	Yes	Yes	Yes	Yes	Yes	Yes	Yes
Study question well-defined anywhere in the article	Yes	Yes	Yes	Yes	Yes	Yes	Yes	Yes	Yes
Placebo control	No	Yes	No	No	No	No	No	No	Yes
Appropriate outcome studied	Yes	Yes	Yes	Yes	Yes	Yes	Yes	Yes	Yes
Multicenter study	Yes	Yes	No	Yes	Yes	Yes	No	Yes	Yes
Study country	China	Argentina	Mexico	Greece	Italy	Iraq	India	USA	Argentina
Adequate selection criteria	Yes	Yes	Yes	Yes	Yes	Yes	Yes	Yes	Yes
Randomization methods described	Yes	Yes	N/A	N/A	No	Yes	No	N/A	Yes
Central randomization site	Yes	Yes	N/A	N/A	No	No	N/D	N/A	N/D
Allocation concealment	N/D	Yes	N/D	N/D	N/D	N/D	No	N/D	Yes
Patients blinded	N/D	Yes	N/D	N/D	N/D	N/D	No	N/D	Yes
Caregivers blinded	N/D	Yes	N/D	N/D	N/D	N/D	No	N/D	Yes
Outcome assessors blinded	N/D	N/D	N/D	N/D	N/D	N/D	N/D	N/D	Yes
Data analysts blinded	N/D	N/D	N/D	N/D	N/D	N/D	N/D	N/D	No
Double blinded	N/D	Yes	N/D	N/D	N/D	N/D	No	N/D	Yes
Vital statistical measures	No	Yes	No	Yes	No	No	Yes	Yes	Yes
Statistician author or acknowledged	Yes	N/D	No	No	Yes	No	No	No	Yes
Intention-to-treat analysis	Yes	Yes	No	No	Yes	No	No	No	Yes
Power calculation reported	No	Yes	No	No	No	No	No	No	Yes
Stopping rules described	No	Yes	No	No	No	No	No	No	No
Baseline characteristics reported	Yes	Yes	Yes	Yes	Yes	Yes	Yes	Yes	Yes
Groups similar at baseline	Yes	No	N/A	N/A	Yes	Yes	N/D	Yes	No
Confounders accounted for	Yes	Yes	Yes	Yes	Yes	No	No	Yes	Yes
Percentage dropouts	N/A	Yes	N/A	N/A	N/A	N/A	N/A	N/A	N/A
Reasons for dropout given	N/A	Yes	N/A	N/A	N/A	N/A	N/A	N/A	N/A
Findings support conclusion	Yes	Yes	Yes	Yes	Yes	Yes	Yes	Yes	Yes

*N/A, not available/not applicable; N/D, not defined*.

**Table 2B:** Risk-of-bias assessment of the trials included in the study.

**References**	**Sequence generation risk of bias**	**Allocation concealment risk of bias**	**Selective reporting risk of bias**	**Other sources of risk of bias**	**Blinding participants and personnel risk of bias**	**Blinding outcome assessors' risk of bias**	**Incomplete outcome data risk of bias**
Abolgashemi et al. (27)	High	High	Low	Low	High	High	Low
Agarwal et al. (28)	Low	High	Low	Low	High	High	Low
AlQahtani et al. (60)	Low	High	Low	Low	High	Unclear	Low
Avenado-Sola et al. (62)	Unclear	High	Low	Low	High	High	Low
Bajpai et al. (59)	Low	High	Low	Low	High	High	Low
Donato et al. (30)	High	High	Low	Low	High	High	Low
Erkurt et al. (52)	High	High	Low	Low	High	High	Low
Hartman et al. (32)	High	High	Low	Low	High	High	Low
Gharbaran et al. (31)	Low	High	Low	Low	High	High	Low
Joyner et al. (12)	High	High	Low	Low	High	High	Low
Li et al. (35)	Low	Moderate	Low	Low	Low	Low	Low
Libster et al. (58)	Low	Low	Low	Low	Low	Unclear	Low
Olivares-Gazca et al. (38)	High	High	Low	Low	High	High	Low
Pappa et al. (39)	High	High	Low	Low	High	High	Low
Perotti et al. (41)	Low	Low	Low	Low	Low	Low	Low
Rasheed et al. (42)	High	High	Low	Low	High	High	Low
Ray et al. (61)	High	High	Low	Low	High	Unclear	Low
Salazar et al. (43)	High	High	Low	Low	High	High	Low
Simonovich et al. (56)	Low	Low	Low	Low	Low	Low	Low

**Table 2C:** Quality assessment for case–control studies included in the study.

**References**		**Altuntas et al. (54)**	**Duan et al. (11)**	**Hegerova et al. (33)**	**Liu et al. (35)**	**Xia et al. (47)**	**Zeng et al. (49)**
Selection	Case definition	–	*	*	*	–	–
	Representativeness of cases	*	-	*	*	*	*
	Selection of controls	*	*	*	*	*	*
	Definition of controls	*	*	*	*	*	*
Comparability of cohorts		**	**	**	**	–	–
Exposure	Ascertainment of exposures	*	*	*	*	*	*
	Same method for both groups	*	*	*	*	*	*
	Non-response rate	*	–	–	–	–	–
Total number of stars		8/9	7/9	8/9	8/9	5/9	5/9

*Note: A study can be awarded a maximum of one star for each numbered item within the Selection and Exposure categories. A maximum of two stars can be given for Comparability*.

**Table 2D:** Quality assessment for cohort studies included in the study.

**References**		**Maor et al. (36)**	**Omrani et al. (53)**	**Rogers et al. (55)**	**Yoon et al. (57)**
Selection	Representativeness of cohort	*	*	*	*
	Selection of non-exposed cohort	–	*	*	*
	Ascertainment of exposure	*	*	*	*
	Outcome not present at the beginning	–	*	*	*
Comparability of cohorts		–	**	**	**
Outcome	Assessment of outcome	*	*	*	*
	Follow-up length	**-**	*	**-**	*
	Adequacy of follow-up	*	*	*	*
Total number of stars		4/9	9/9	8/9	9/9

*Note: A study can be awarded a maximum of one star for each numbered item within the Selection and Exposure categories. A maximum of two stars can be given for Comparability*.

**Table 2E:** Quality assessment of the case studies included in the study.

**References**	**Ahn et al. (29)**	**Karekadavath et al. (51)**	**Martinez-Resendez et al. (37)**	**Pei et al. (40)**	**Shen et al. (44)**	**Tan et al. (45)**	**Wang et al. (46)**	**Ye et al. (48)**	**Zhang et al. (50)**
1. Was the study question or objective clearly stated?	Yes	Yes	Yes	Yes	Yes	Yes	Yes	Yes	Yes
2. Was the study population clearly and fully described, including a case definition?	Yes	No	Yes	Yes	Yes	Yes	No	Yes	Yes
3. Were the cases consecutive?	No	Yes	N/D	N/D	No	N/D	N/D	No	No
4. Were the subjects comparable?	Yes	Yes	Yes	Yes	Yes	No	Yes	No	Yes
5. Was the intervention clearly described?	Yes	Yes	Yes	No	Yes	No	Yes	Yes	Yes
6. Were the outcome measures clearly defined, valid, reliable, and implemented consistently across all study participants?	Yes	Yes	Yes	No	Yes	No	Yes	Yes	Yes
7. Was the length of follow-up adequate?	Yes	Yes	No	Yes	Yes	No	No	No	Yes
8. Were the statistical methods well-described?	N/A	N/A	Yes	N/A	N/A	N/A	Yes	N/A	N/A
9. Were the results well-described?	Yes	Yes	Yes	Yes	Yes	Yes	Yes	Yes	Yes
Quality rating	Good	Good	Good	Fair	Good	Poor	Fair	Fair	Good

*N/A, not applicable; N/D, not defined*.

## Discussion

In this systematic review and meta-analysis of CPT in COVID-19 patients, 38 studies (11, 12, 27–62) were included and critically evaluated. All included studies reported excellent outcomes for CPT in COVID-19. Our systemic review and meta-analysis is one of the first ones to summarize all such existing evidence on the efficacy and safety of CPT in humans with COVID-19. According to the results of our systematic review and meta-analysis, CPT is effective in reducing the mortality rate and has low incidence of serious adverse events during and after convalescent plasma infusion, which are mostly controllable.

CPT confers immediate immunity via interruption of the viral entry into the cells. Additionally, in the context of COVID-19, neutralizing antibodies are anticipated to be the primary active agent in convalescent plasma and the marker of plasma potency (9). In the past, CPT has been shown to provide benefits in severe acute respiratory syndromes (6). Prior studies have also reported promising outcomes in Spanish influenza A (H1N1) infection (63), avian influenza A (H5N1) (64), viral hemorrhagic fevers such as Ebola (65), influenza A (H1N1) infections in 2009/2010 (66), and SARS-CoV infections in 2003 (67). A systematic review and meta-analysis revealed a consistent reduction in mortality with the use of plasma therapy (6). The results are similar to our findings. One of the possible hypotheses for the observed decreased mortality could be due to antibodies that can hamper virus reproduction in the active phase of infection and help clear the virus, which is advantageous to the rapid recovery of the disease (67). Mechanistic and clinical data also support the observed mortality reduction benefit associated with convalescent plasma administration (68, 69).

There was no significant reduction in mortality rate between patients with CPT and controls based on data from RCTs. However, sensitivity analysis [excluding the study by Agarwal et al. (28)] revealed that patients transfused with CPT had a lower mortality rate. The Agarwal et al. (28) trial comprised ~70% of the patients in the CPT cohort who received plasma with low levels of SARS-CoV-2 antibodies. Additionally, the remaining 30% of the patients received plasma with no detectable antibodies. Thus, there were strong methodical and clinical rationales to exclude this study from statistical models during sensitivity analysis. Nevertheless, Agarwal et al. (28) did observe a positive effect of CPT on clinical symptoms and viral clearance.

It is worth noting that the doses of CPT vary between the included studies. However, the Chinese study (11) described the use of a single dose of 200 ml of convalescent plasma, whereas Bin Zhang et al. (50) reported a maximum of 2,400 ml of convalescent plasma. The optimal dose of CPT for COVID-19, therefore, could not be estimated.

It is also important to note that the included patients were critically ill and received ICU admission (*n* = 12,095) or underwent mechanical ventilation (*n* = 8,200) and that all COVID-19 patients described in our meta-analysis received concomitant antiviral drugs and steroids including CPT; also, many patients received antibacterial/antifungal drugs for co-infection. All included studies described little mortality with the use of CPT, and the pooled analysis suggests a mortality rate of 12.9% (95% CI 9.7–16.9). However, the individual impact of CPT could not be determined as patients also received multiple other agents (including antiviral medications). Therefore, further studies evaluating the use of CPT alone are warranted.

The safety profile of CPT in COVID-19 has not been described in detail. The observed pooled adverse event rate was 6.1% (95% CI 3.2–11.6). This suggests that CPT was well-tolerated by the participants in the included studies. It is important to note that no fatality was reported as adverse event with the use of CPT. Human plasma transfusion is routinely performed in hospitals. Human anti-SARS-CoV-2 plasma differs from standard plasma as it contains antibodies against SARS-CoV-2. The risks to transfusion recipients are similar to those of standard plasma. The risk of transfusion-transmissible infection is low in developed countries. The incidence rates of infections such as HIV, hepatitis B, and hepatitis C are less than one infection per 2 million donations (70). Other adverse events with plasma therapy include allergic transfusion reactions, transfusion-associated circulatory overload (TACO), and transfusion-related acute lung injury (TRALI) (71). Even though TRALI occurs in <1 for every 5,000 transfused units, it is concerning in COVID-19 patients. Donor screening including HLA antibody screening decreases the risk of TRALI (72).

A risk benefit analysis based on age, symptoms, comorbidities, and COVID-19 transmission parameters was published in a recent review by Bloch et al. (73). Five hundred simulations were carried out, assuming varying degrees of effectiveness of convalescent plasma treatment. The model revealed that convalescent plasma was beneficial in COVID-19 infection even at the lowest estimates of 25% effectiveness. In other words, the model suggests that the potential benefit of plasma therapy outweighs the risk of transfusion in COVID-19 infection (73).

The important strengths of our study are a comprehensive search of the already published clinical studies and the large number of patients included in the analysis. This is one of the first meta-analyses on CPT use in COVID-19 patients showing an overwhelming positive result. The review of sample of articles by two co-authors is again a testimony of the quality check of data collection in this review. The generalizability of these results is also a strength of this article.

Despite the numerous strengths of the meta-analysis, there are certain limitations. One of the limitations of the meta-analysis is integral to the methodology. The summarization of varying pieces of information may ignore important differences between studies. Nonetheless, this is a controversial aspect of the meta-analysis (74). Additionally, a meta-analysis generalizes results despite differences in primary research and does not simply report a summary effect. The heterogeneity is high in our studies, especially regarding the pooled adverse event rate and pooled mortality rate. Further studies may be needed to confirm the findings and explain the mechanisms. A lack of high-quality RCT studies and relevant literature paucity limited our analyses. All the reported studies were predominately case reports or series, had no proper control groups, and had a moderate to high risk of bias. Most studies in our meta-analysis were observational studies with a high risk of bias, which are subject to inherent limitations of the study design with unmeasured differences in the study population and residual confounders despite all adjustments. The currently available evidence on the safety and effectiveness of convalescent plasma for treatment of people with COVID-19 is of very low strength. Our study predominantly describes the clinical data and incidence rates in hospitalized patients. Also, we could not register the review. We tried to prospectively register our systematic review but decided to go against it as it was taking an unreasonably longer time than expected due to the increased pool of COVID-19-related articles. Another limitation of our study is the inclusion of 12 studies (30, 31, 37, 39, 40, 42, 45, 57–59, 61, 62) from the preprint databases which have not been peer reviewed and are necessary for a thorough evaluation of the currently available data on CPT in our meta-analysis. Preprint articles possibly indicate the undetermined quality of available literature and biased articles on CPT; however, we will update the status of these above-mentioned studies in the risk-of-bias table. Lastly, many studies were determined to have a significant risk of bias. This was related to a combination of factors such as non-randomized design, confounding, poor methodological conduct, and limited information on dose and duration of the CPT. Importantly, many of the patients enrolled in the studies included in the present analysis received convalescent plasma transfusions later in their disease course. As a result, our analysis may underestimate the mortality reduction achievable through early administration of high-titer convalescent plasma for COVID-19. Based on low-quality evidence, there is no suggestion that convalescent plasma would cause any serious adverse events in patients with COVID-19 and lower the mortality in COVID-19 patients. Thus, any conclusions that are drawn based on these data are of limited value, and these conclusions are subject to change as more reliable results become available.

## Conclusion

Based on the consolidated clinical data derived from the systemic review and meta-analysis, it is suggested that, in addition to antiviral/antimicrobial drugs and steroids, CPT could be an effective concomitant therapeutic option as the use of CPT decreased mortality with a safe clinical profile and promising evidence on the safety and reduced mortality. We recognize that a definitive conclusion cannot be drawn regarding optimal doses and treatment time point for the CPT. Future larger observational studies (75) and clinical trials could benefit from more standardized reporting, especially in terms of the timing of intervention and clinically relevant outcomes, like days until discharge from hospital and improvement of clinical symptoms.

## Data Availability Statement

The raw data supporting the conclusions of this article will be made available by the authors, without undue reservation.

## Author Contributions

Data review and collection were done by KM, IG, SG, RS, AT, PA, HK, and SD. Statistical analysis was done by VB, AB, and MS. Study design and distribution of articles for critical review were done by IM and RK. VB and KM are the guarantors of the paper, taking responsibility for the integrity of the work as a whole, and from inception to publication of the article. Final approval was given by all authors. All authors contributed to the article and approved the submitted version.

## Conflict of Interest

The authors declare that the research was conducted in the absence of any commercial or financial relationships that could be construed as a potential conflict of interest.

## Abbreviations

COVID-19: Coronavirus Disease 2019
CPT: immunotherapy with convalescent plasma
FDA: Food and Drug Administration
HIV: human immunodeficiency virus
RNA: ribonucleotide acid
SARS-CoV-2: Severe Acute Respiratory Syndrome Coronavirus 2
TRALI: transfusion-related acute lung injury
WHO: World Health Organization.
